# Facial Gunshot Wound: Mandibular Fracture With Internal Fixation and a Pectoralis Myocutaneous Flap Coverage

**DOI:** 10.7759/cureus.14214

**Published:** 2021-03-31

**Authors:** William Aukerman, Michaela Hull, Siddhartha Nannapaneni, Kamran Shayesteh

**Affiliations:** 1 Surgery, Conemaugh Memorial Medical Center, Johnstown, USA; 2 Surgery, Lake Erie College of Osteopathic Medicine, Erie, USA; 3 Plastic and Reconstructive Surgery, Conemaugh Memorial Medical Center, Johnstown, USA

**Keywords:** gunshot, mandible fracture, pectoralis myocutaneous flap

## Abstract

Facial penetrating gunshot wounds (GSWs) are seen in an assault, suicide, and accidental injury. They often carry high mortality given the important anatomical structures located within the neck. The foundations of maxillofacial GSWs are rooted in data from military combat, specifically the last world war. This type of injury is complex for reconstructive surgery due to significant soft tissue and bone loss. Management of maxillofacial GSWs is often challenging and has trended from serial debridement, immediate reconstruction, local tissue flaps, and distant free flap transfers depending on bullet trajectory and wound intricacy.

We present a case of a 51-year-old male with a 22-caliber GSW to the left side of his face. Hemodynamics were stable on arrival and history included alcohol use. A left mandibular wound measured approximately 8 cm in diameter with exposed bone. A small 0.5-1 cm wound was also present inferiorly. A maxillofacial CT scan was utilized, showing a left mandibular body fracture.

The patient underwent exploration and debridement on the same day of injury. Open reduction with internal fixation of the left mandible fracture and Synthes 2.5 mm locking plate was done. Additionally, a left pectoralis major myocutaneous muscle flap was performed two days later.

Regional pectoralis flap reconstruction of facial firearm injury is scarcely acknowledged in the literature. Due to the location of the wound, the functionality of the jaw can be maintained in addition to ample blood supply by performing mandibular fixation and pectoralis major myocutaneous flap.

## Introduction

Facial penetrating gunshot wounds (GSWs) are seen in an assault, suicide, and accidental injury. Approximately 32,000 deaths per year and 67,000 injuries are caused by firearms each year [1]. A retrospective study showed nearly 6% of 4,100 GSWs involved the face and can be further classified into penetrative, avulsive, and perforative [2,3]. This type of facial trauma is complex for reconstructive surgery due to significant soft tissue and bone loss from high-velocity impact. Initial stabilization, definitive reconstruction, and secondary refinement make up the mainstay of treatment while immediate, early, or delayed treatment remains a controversial topic [3]. Management of facial GSWs has trended from serial debridement, immediate reconstruction, local tissue flaps, and distant free flap transfer depending on bullet trajectory and wound intricacy [2,4,5]. Added complexity comes from further consideration of disputed management and long-term postoperative follow-up.

## Case presentation

**Figure 1:**
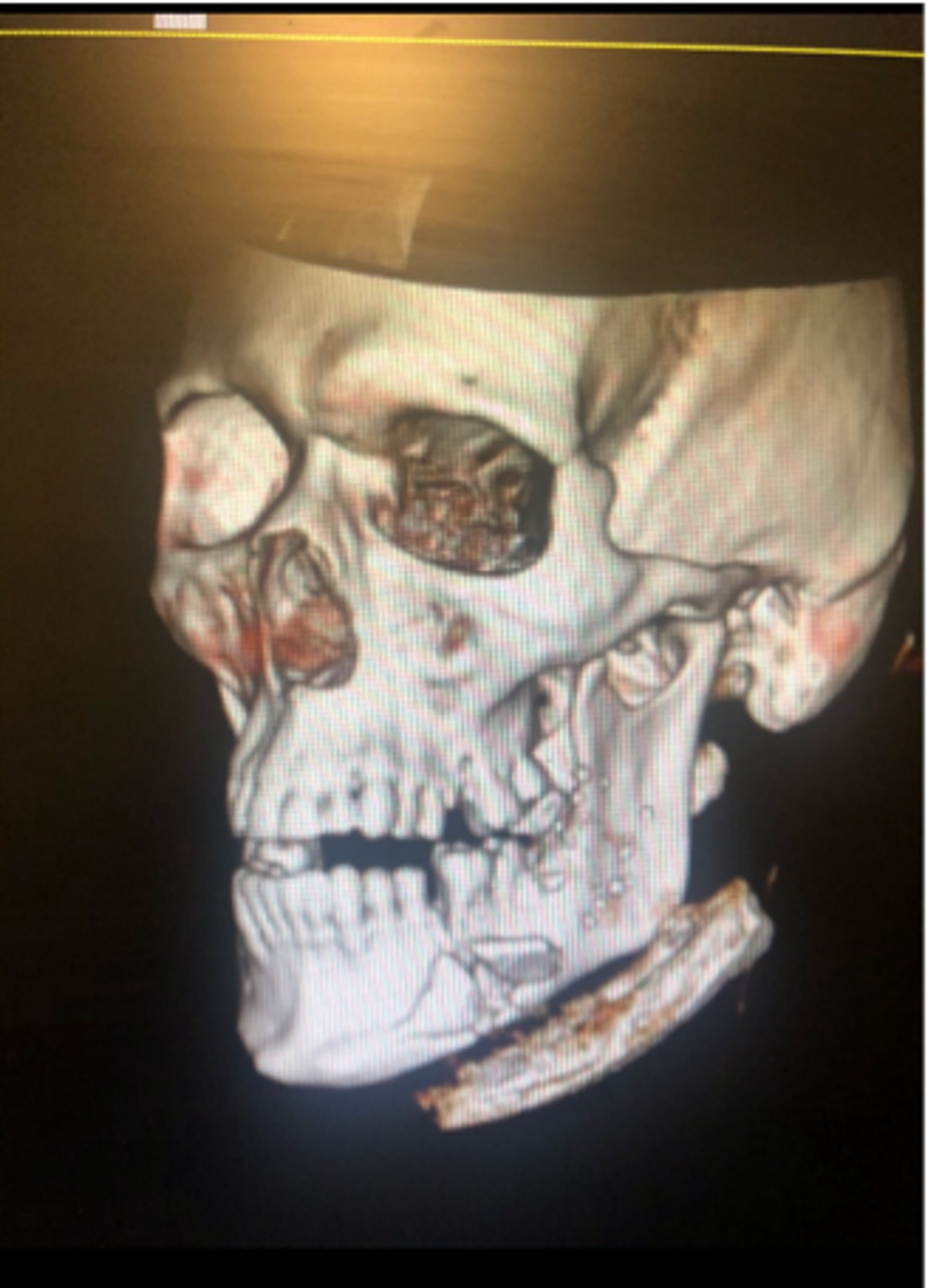
Maxillofacial CT scan demonstrating left mandible fracture.

We present a case of a 51-year-old male who presented to our Level 1 Trauma center, with a 22-caliber self-inflicted penetrating GSW to his left mandible. On presentation, the patient was hemodynamically stable, however, elicited inconsistent reports regarding the history of his GSW. Past medical history revealed alcohol abuse and tobacco use. The patient was found to have a blood alcohol level of 0.182 upon arrival. On primary survey, both the bullet entry and exit sites were visualized on the left side of his face. The open exit wound was measured at 6 x 8 cm extending down to the mandible. A small 0.5-1 cm entry wound was also present on the left side of his chin inferiorly. A maxillofacial CT scan was utilized to make a diagnosis of left-sided mandibular body fracture (Figure 1). 

**Figure 2:**
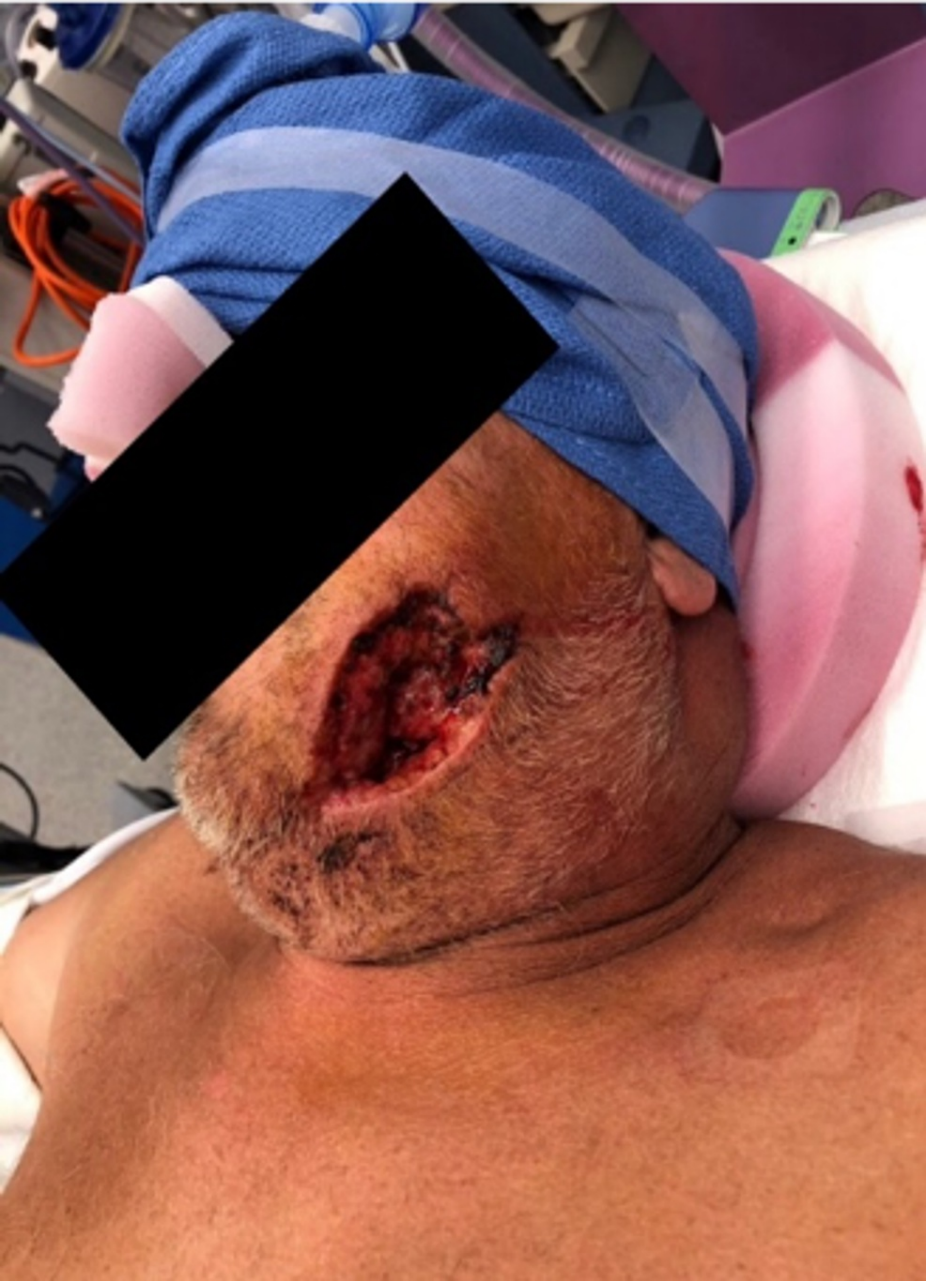
Preoperative evaluation of the extent of injury (left mandible).

Due to full-thickness skin loss, subcutaneous tissue, and muscle with extensive exposure to the mandible, our Plastic and Reconstructive Surgery team was consulted (Figure 2).

**Figure 3:**
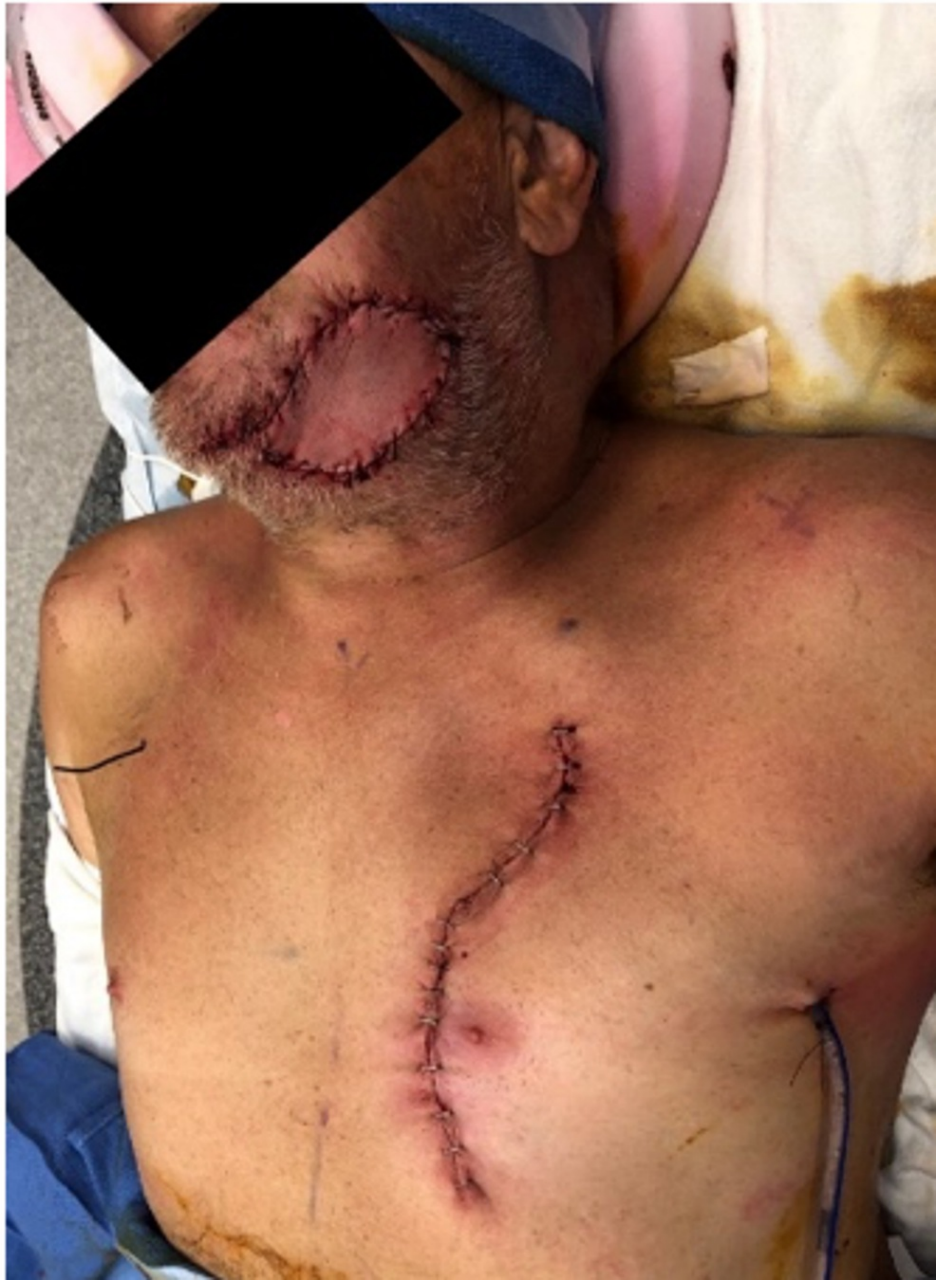
Postoperative left pedicled myocutaneous pectoralis flap.

The patient was further evaluated and subsequently underwent exploration and debridement during post-trauma day one. Multiple comminuted fragments of bone were debrided and removed at that time. The following day, the patient underwent mandibulomaxillary fixation with Synthes matrix wave, including open reduction and internal fixation (ORIF) of the left mandible fracture with a Synthes 2.5 mm locking plate. In addition, a left pectoralis major myocutaneous muscle flap with subcutaneous tunneling was performed. A 6 x 8 cm skin pedicle medial to the nipple was dissected to the level of the pectoralis major muscle. The pedicle myocutaneous flap was rotated and passed through a subcutaneous tunnel and above the clavicle to the facial defect (Figure 3).

The patient tolerated the procedure well and was transferred to the floor in stable condition. Dental arch bars were removed on postoperative day 14. At that time, the patient was tolerating a liquid diet and had no complaints of pain or discomfort in regards to speaking or opening/closing of his jaw. He was discharged post-op day 15 with a scheduled follow-up appointment with his primary care physician and was compliant with follow up in the Plastic and Reconstructive Surgery clinic.

## Discussion

The initial treatment of penetrating neck and maxillofacial trauma can be challenging and is of continued debate. A timely diagnosis and examination of the injury extent are crucial in order to prevent possible tissue necrosis, ischemia, and infection. Early staged procedures, including debridement and fixation, have been advocated [2]. Subsequent definitive reconstruction can aid in improved quality of life and functionality. 

Even though the foundations of maxillofacial GSWs are noted throughout history, there has been scarce documentation of the use of a regional pedicled myocutaneous pectoralis flap reconstruction in regards to these wounds [4]. Today the percentage of nonfatal firearm assaults is steadily increasing, as fatal firearm injuries decrease [1]. Pectoralis major myocutaneous flap reconstruction has been shown to safely reconstruct high-risk patients needing large volume tissue salvage in oncologic defects, however more studies are needed to address open penetrating facial GSWs [6,7]. By performing local tissue flap reconstruction, it is shown that functionality of the jaw can be maintained in addition to ample bloody supply [5,6,8].

## Conclusions

It is evident that providing prompt evaluation and reconstruction, is crucial to increasing the chances of survival and quality of life. Initial management can be challenging and has been of continued debate. In this present report, we demonstrated a case of a patient with a successful outcome with intact function and vascular supply status post subcutaneous tunneled myocutaneous pectoralis major pedicled flap due to a self-inflicted GSW. Regional pedicle myocutaneous flap reconstruction should be more readily considered in large tissue defects due to firearm injuries.
